# Oncometabolite induced primary cilia loss in pheochromocytoma

**DOI:** 10.1530/ERC-18-0134

**Published:** 2018-09-05

**Authors:** Samuel M O’Toole, David S Watson, Tatiana V Novoselova, Lisa E L Romano, Peter J King, Teisha Y Bradshaw, Clare L Thompson, Martin M Knight, Tyson V Sharp, Michael R Barnes, Umasuthan Srirangalingam, William M Drake, J Paul Chapple

**Affiliations:** 1 William Harvey Research InstituteBarts and the London School of Medicine, Queen Mary University of London, London, UK; 2 Department of EndocrinologySt Bartholomew’s Hospital, Barts Health NHS Trust, London, UK; 3 Institute of Bioengineering and School of Engineering and Material SciencesQueen Mary University of London, London, UK; 4 Barts Cancer InstituteQueen Mary University of London, London, UK; 5 Department of Diabetes and EndocrinologyUniversity College London Hospital, London, UK

**Keywords:** pheochromocytoma, primary cilia, hypoxia, succinate dehydrogenase, von Hippel–Lindau protein

## Abstract

Primary cilia are sensory organelles involved in regulation of cellular signaling. Cilia loss is frequently observed in tumors; yet, the responsible mechanisms and consequences for tumorigenesis remain unclear. We demonstrate that cilia structure and function is disrupted in human pheochromocytomas – endocrine tumors of the adrenal medulla. This is concomitant with transcriptional changes within cilia-mediated signaling pathways that are associated with tumorigenesis generally and pheochromocytomas specifically. Importantly, cilia loss was most dramatic in patients with germline mutations in the pseudohypoxia-linked genes *SDHx* and *VHL*. Using a pheochromocytoma cell line derived from rat, we show that hypoxia and oncometabolite-induced pseudohypoxia are key drivers of cilia loss and identify that this is dependent on activation of an Aurora-A/HDAC6 cilia resorption pathway. We also show cilia loss drives dramatic transcriptional changes associated with proliferation and tumorigenesis. Our data provide evidence for primary cilia dysfunction contributing to pathogenesis of pheochromocytoma by a hypoxic/pseudohypoxic mechanism and implicates oncometabolites as ciliary regulators. This is important as pheochromocytomas can cause mortality by mechanisms including catecholamine production and malignant transformation, while hypoxia is a general feature of solid tumors. Moreover, pseudohypoxia-induced cilia resorption can be pharmacologically inhibited, suggesting potential for therapeutic intervention.

## Introduction

Pheochromocytomas (PCCs) are neuroendocrine tumors that originate from chromaffin cells of the adrenal medulla or autonomic nervous system, where they are termed paragangliomas (PGLs). The majority of the morbidity associated with PCC/PGLs is consequent upon their production of catecholamines, leading to severe, life-threatening hypertension, but they may also cause local mass effect and have the potential for metastatic spread (Fishbein & Nathanson 2012, Burnichon *et al.* 2016). Understanding of the pathogenesis of PCC/PGLs is incomplete, with limited ability to predict malignant potential and at present the response to conventional cancer therapies is disappointing.

Approximately 30% of PCC/PGLs are associated with inherited germline mutations in more than 15 different susceptibility genes (Dahia 2017). These include causative genes for inherited cancer syndromes, where, relative to other tumor types, there is a high incidence of PCC/PGL. Recent analyses of germline and somatic mutations have classified PCC/PGL into four molecularly defined groups, including a pseudohypoxia-linked subtype (Fishbein *et al.* 2017). These pseudohypoxic tumors occur due to mutations that impact regulation of the hypoxia transcription factors HIF1α and HIF2α. This can be through germline or somatic mutation of the ubiquitin E3 ligase pVHL (von Hippel–Lindau protein), which targets HIFα for degradation by the ubiquitin proteasome system (Dannenberg *et al.* 2003, Gossage *et al.* 2015, Crespigio *et al.* 2017). Increased HIF activity also results from germline mutation in genes that encode the succinate dehydrogenase (SDH) complex subunits (SDHA, SDHB, SDHC, SDHD), succinate dehydrogenase complex assembly factor 2 (SDHAF2), fumarate hydratase (FH) and malate dehydrogenase (MDH2) (Fishbein & Nathanson 2012). This is because loss of their function leads to accumulation of oncometabolites that inhibit pVHL-mediated degradation of HIFα (Selak *et al.* 2005).

Although pseudohypoxic mechanisms account, at least in part, for angiogenesis-facilitated growth, they do not, of themselves, satisfactorily explain PCC/PGL tumorigenesis. Mutations in the *VHL* gene are known to be important in renal cancers; this includes the occurrence of clear cell renal cell carcinoma (ccRCC) as part of the inherited cancer syndrome von Hippel–Lindau disease, in which *VHL* is mutated and PCC/PGL can occur (Gossage *et al.* 2015, Crespigio *et al.* 2017). One of the hallmark features of ccRCC is the loss of primary cilia (Basten *et al.* 2013), which act as flow sensors on renal epithelial cells. Cilia are cellular organelles that consist of a microtubule-based core structure, known as the axoneme, which elongates from a basal body and is covered by the ciliary membrane. Cilia function as signaling platforms involved in the transduction of extracellular stimuli, through mechanisms including regulating the spatial compartmentalization of signaling components (Berbari *et al.* 2009, Goetz & Anderson 2010). For example, primary cilia are modulators of WNT signaling and have an essential role in mammalian hedgehog (Hh) signaling (Berbari *et al.* 2009, Wong *et al.* 2009, Goetz & Anderson 2010, Lancaster *et al.* 2011, Oh & Katsanis 2013).

The coordination of cilia-mediated signaling is influenced by the dynamic nature of cilia, which elongate and shorten in response to cell cycle stage and other stimuli. This requires the process of intraflagellar transport (IFT) to traffic ciliary components in both anterograde and retrograde directions along axonemal microtubules. Cilia are assembled when cells enter stationary phase and are normally resorbed prior to cell division. This occurs as the basal body, which acts as a nucleation site for the growth of axoneme microtubules during ciliogenesis, is derived from a mother centriole and is required for mitotic spindle pole formation. Importantly, the mother centriole has this dual role means that the presence of a primary cilium potentially acts as a checkpoint within the cell cycle (Izawa *et al.* 2015). Thus, cilia might oppose cell division and proliferation; however, it should be noted that there are instances where cilia are present on mitotic cells (Goto *et al.* 2013). Dysregulation of normal restraints on cellular proliferation is required for neoplastic progression, and it is hypothesized that disruption of a ciliary cell cycle checkpoint may promote tumorigenesis (Mans *et al.* 2008), although ciliopathy patients have not been identified as having an increased risk of cancer (Johnson & Collis 2016).

Here, we address key questions regarding the loss of cilia in tumor cells in the context of PCC/PGLs. These include whether cilia loss is correlated with changes in cilia-mediated signaling *in vivo.* We also consider whether cilia loss increases cellular proliferation or is a consequence of it. We demonstrate that primary cilia loss is a feature of PCC/PGL and in particular those that are driven by germline mutations in pseudohypoxia-linked genes. This finding is consistent with transcriptome-based evidence from PCCs for dysregulation of cilia maintenance and cilia-mediated signaling pathways. Using a rat PCC-derived cell line (PC12), we define the molecular mechanism of primary cilia loss, demonstrating that axonemal resorption is dependent on both HIF signaling and Aurora-A kinase activation. Moreover, loss of primary cilia in PC12, induced by ciliary protein knockdown, leads to increased proliferation and alterations in expression of genes associated with pathways involved in proliferation and cancer. We also show that knockdown of pseudohypoxia-causing PCC/PGL genes and treatment with inhibitors that trigger accumulation of oncometabolites result in primary cilia loss.

## Materials and methods

### Tissue sample collection and preparation for immunolabeling and RNA extraction

Samples of tumor and adjacent adrenal medulla, where available, were collected at the time of adrenalectomy (for PCC) or PGL resection (patient recruitment and ethical approval is described in the Supplementary data, see section on supplementary data given at the end of this article). PCC and normal adrenal medulla were differentiated at the time of surgery with subsequent pathology analysis. For immunofluorescence, samples were fixed in 4% paraformaldehyde, resuspended in 30% sucrose and embedded in OCT compound (VWR) prior to storage at −80°C. For RNA extraction, tissue samples were placed directly into RNAlater (Thermo Fisher Scientific) and stored at −20°C. Samples were subsequently homogenized in RLT buffer and purified using RNeasy Mini Kit (Qiagen).

### Cell culture and experimental treatments

Cell lines were cultured and treated with drugs as described in Supplementary data. PC-12 Adh (ATCC CRL-1721.1) cells were obtained from the American Type Culture Collection; for cilia assembly experiments, cells were plated and grown in complete media for 24 h prior to serum starvation for a further 24 h or otherwise specified. For cilia disassembly experiments serum-containing media was reintroduced for 24 h after starvation or as otherwise specified.

### Immunofluorescent detection and quantification of primary cilia

The immmunostaining protocols and antibodies used are described in Supplementary data. Confocal microscopy was performed using an LSM510 or LSM880 laser scanning confocal microscope (Zeiss). Quantification of cilia incidence and length was performed blinded to experimental status. Cilia incidence was defined as the number of cells with a cilium (identified by two axonemal markers) divided by the number of nuclei in a given field. Cilia length was measured from maximum intensity projections created from confocal Z-stacks using Zen (Zeiss) and ImageJ (NIH) software. The surpass module of Imaris 7.1 image processing and analysis software (Bitplane) was used to surface render 3D images.

### siRNA-mediated knockdown

PC12 cells were transfected with either targeted or non-targeted control siRNAs (Silencer Select, Ambion) using Lipofectamine 3000 (Thermo Fisher Scientific), according to the manufacturer’s instructions. For knockdown of *VHL*, *SDHB* and *FH*, *IFT88* and *Cep164* – two distinct siRNAs each targeting distinct exons – were used at a total concentration of 30 nM (sequences available on request).

### RNA-sequence data and pathway analyses

RNA extraction and sequencing is described in Supplementary data. All analyses were conducted in the R statistical environment, version 3.4.0, using software from the Bioconductor repository (Huber *et al.* 2015). Functional analysis of differential gene expression between control and *Ift88*-knockdown cells was performed using Ingenuity Pathways Analysis (IPA; Ingenuity Systems), using all genes with log fold change ≥2 and *q*-value was <0.01, as input. For all gene set enrichment analyses, a right-tailed Fisher’s exact test was used to calculate a pathway *P*-value determining the probability that each biological function assigned to that data set was due to chance alone. All enrichment scores were calculated in IPA using all transcripts that passed QC as the background data set. For more details of transcriptome and pathway analyses, see our supplemental R Markdown document (https://github.com/C4TB/markdown-chapple_pcc).

## Results

### The incidence and length of primary cilia is reduced in PCCs relative to adjacent normal adrenal medulla

**Figure 1:**
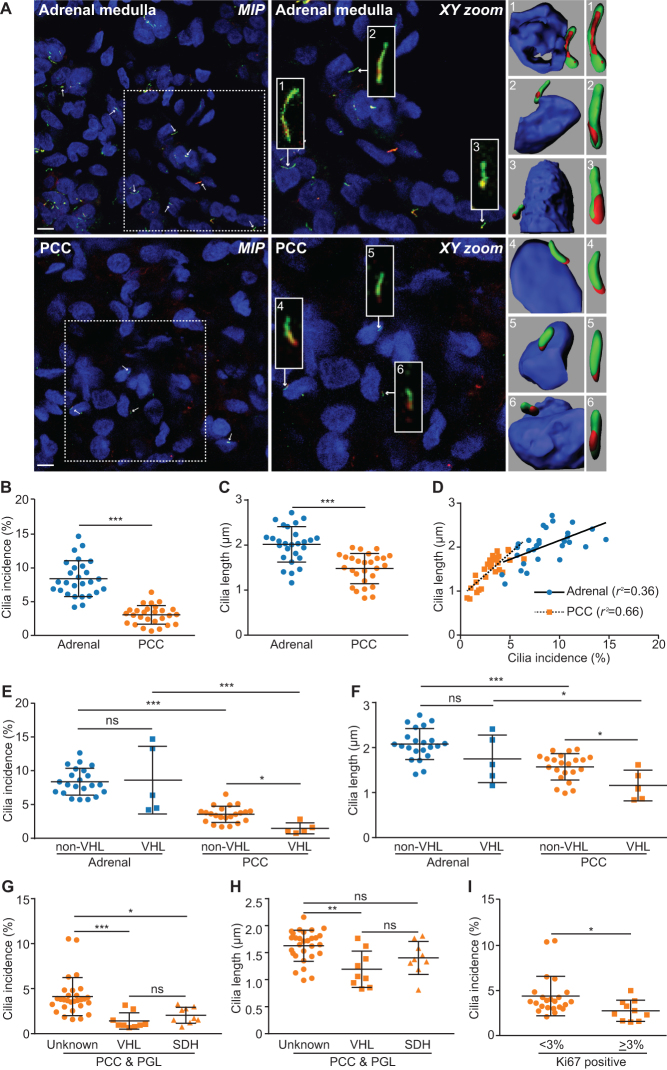
Primary cilia incidence and length is reduced in PCCs relative to adjacent adrenal medulla. (A) Maximum intensity projections (*MIP*) of confocal Z-stacks of PCC and adjacent adrenal medulla. Tissue sections were processed for dual-immunofluorescent detection of the ciliary markers acetylated α-tubulin (green) and Arl13b (red). They were then counterstained with DAPI (blue) to detect nuclei. A single confocal section from the area demarked by the dashed box is shown zoomed (*XY zoom*). Individual cilia, indicated by arrows, are further enlarged in insets 1–6 and are shown as surface rendered 3D images in the panels on the right. Scale bars = 10 µm. (B) Quantification of primary cilium incidence in 27 paired PCC and adjacent adrenal medulla tissue samples. (C) Quantification of axoneme length (from confocal Z-stacks) from cells that had a primary cilium in PCC and adjacent adrenal medulla. (D) Cilia incidence and length correlate in both PCCs and adjacent adrenal medulla, with a more significant relationship in tumor than normal tissue. (E and F) Cilia incidence and length in 27 paired PCCs and adrenal medulla samples comparing individuals with (*n* = 5) and without (*n* = 22) germline mutations in VHL. (G and H) Cilia incidence and length in 47 PCC/PGL comparing those with a germline mutations in VHL (*n* = 9), to tumors from patients with germline mutations in *SDHx* (*n* = 9) and those without a known germline mutation in a pseudohypoxia-linked gene (Con, *n* = 29). (I) Cilia incidence in 33 PCC where more or less than 3% of cells labeled positively for Ki67. The number of cilia and nuclei were counted in 15 randomly selected fields for each sample. Mean axonemal length was quantified from at least 50 ciliated cells for each sample. Error bars indicate s.d. Statistical tests: *t*-test (B, C, E, F and I), ANOVA (G and H), linear regression (D). *P < 0.05, **P < 0.01, ***P < 0.001.

We collected paired tissue samples from PCCs and adjacent macroscopically normal adrenal medulla from 25 patients who underwent adrenalectomy. Two individuals had bilateral disease giving a total of 27 paired samples (Table 1 and Supplementary Table 1). The tissues were immunostained for the axonemal proteins acetylated α-tubulin and ADP-ribosylation factor-like protein 13B (Arl13b) and analyzed for the incidence of cells with a primary cilium (Fig. 1A). This showed that the occurrence of a primary cilium was lower (*P* = 4.74 × 10^−11^) in PCCs (3.06 ± 0.14% of cells) compared to adrenal medulla (8.42 ± 0.03% of cells) (Fig. 1B). The length of the ciliary axoneme was also reduced (*P* = 8.24 × 10^−11^) in PCC cells that still had cilia (1.48 ± 0.34 μm) relative to cells in adjacent adrenal medulla (2.02 ± 0.39 μm) (Fig. 1C). The incidence and length of primary cilia measured in individuals correlated in both PCCs and adjacent adrenal medulla, although this relationship was stronger in PCCs than adjacent adrenal medulla (PCC *P* < 0.001, *r* ^2^ = 0.66; adrenal *P* = 0.001, *r* ^2^ = 0.36) (Fig. 1D). We also observed that in every instance cilia incidence was lower in the PCC than its adjacent adrenal medulla (Supplementary Fig. 1A). This was also the case for cilia length in all but one of the paired samples (Supplementary Fig. 1B). Together, these data established that loss of primary cilia is a feature of PCC.

**Table 1:** Clinical details – summary table.

	Paired	Unpaired	All
Samples (*n*)	27	20	47
Patients (*n*)	25	15	40
Sex			
Male; *n* (%)	12 (48)	7 (47)	19 (47.5)
Female; *n* (%)	13 (52)	8 (53)	21 (52.5)
Age (years)			
Mean ± s.e.m.	46.8 ± 4.0	46.2 ± 3.4	46.6 ± 2.8
Range	12–78	15–68	12–78
Size (mm)			
Mean ± s.e.m.	49 ± 4	47 ± 7	48 ± 4
Range	8–87	13–120	8–120
Location			
Adrenal (%)	27 (100)	7 (35)	34 (72)
PGL (%)	0 (0)	13 (65)	13 (28)
Mode of diagnosis			
Symptomatic (%)	9 (33)	9 (45)	18 (38)
Incidental (%)	14 (52)	7 (35)	21 (45)
Screening (%)	4 (15)	4 (20)	8 (17)
Germline mutation (patients) (%)	5 (25)	8 (53)	13 (33.5)
SDHA	0	1	1
SDHB	1	4	5
VHL	3	2	5
MEN2	1	1	2
Germline mutation (tumors) (%)	7 (26)	13 (65)	20 (42.6)
SDHA	0	3	3
SDHB	1	5	6
VHL	5	4	9
MEN2	1	1	2

Incidental, diagnosis due to investigation for another unrelated condition; MEN2, multiple endocrine neoplasia 2; mode of diagnosis – symptomatic, diagnosis due to symptoms or signs of catecholamine excess leading to diagnosis; PGL, paraganglioma; screening, diagnosis during a screening program in individuals with known pheo/PGL predisposition; SDH, succinate dehydrogenase; s.e.m., standard error of the mean; VHL, von Hippel–Lindau.

It has previously been reported that the tumor suppressor pVHL plays a role in ciliogenesis (Schermer *et al.* 2006). Thus, we next compared cilia loss and length reduction in PCC from patients with germline mutations in *VHL* compared to those without. We found that both cilia incidence (1.40 ± 0.01% vs 3.43 ± 0.02) and length (1.15 ± 0.35 μm vs 1.58 ± 0.31 μm) were reduced in VHL-PCCs compared to non-VHL-PCCs (*P* = 0.010 for incidence, *P* = 0.010 for length) (Fig. 1E and F). There was no significant difference in either cilia incidence or length in adjacent adrenal medulla from *VHL* and non-*VHL* patients (Fig. 1E and F). This suggests that cilia loss and shortening in VHL-PCCs occurs during tumorigenesis and is not a pre-existing/pre-malignant feature.

In order to further evaluate whether this finding was specific to VHL or a feature of other pseudohypoxic PCCs, we extended our analysis to include an additional 20 tumors from 15 patients from whom a paired adrenal sample was unavailable (total 47 PCC/PGL from 40 patients; Table 1 and Supplementary Table 2). We compared PCC/PGLs from patients with germline mutations in VHL, to tumors from patients with germline mutations in SDHx and those without a known germline mutation in a pseudohypoxia-linked gene. Cilia incidence was reduced in PCC/PGLs from patients with germline mutations in VHL (*P* = 0.0007) and SDHx (*P* = 0.0103 for incidence), relative to PCC/PGLs from patients that were not of a pseudohypoxia-linked subtype (Fig. 1G). Cilia length was also reduced in VHL- and SDHx-PCC/PGLs relative to the non-pseudohypoxia tumors, although this was only significant for VHL (*P* = 0.0013) (Fig. 1H).

We also examined if there was any correlation between cilia loss and clinical disease parameters in patients with PCC/PGL. Patients under 18 years of age at the time of surgery had tumor cells with fewer and shorter cilia than patients who were over the age of 18 years (Supplementary Fig. 1C and D), suggesting an association between cilia loss and age (at time of surgery). As the presence of a primary cilium is potentially a checkpoint for cell division, we next tested if cilia loss correlated with cellular proliferation in PCC/PGLs. This was by quantifying the percentage of cells that labeled positively for Ki67 (quantified by routine clinical immunohistochemistry), a marker of proliferative activity that has previously been correlated with malignant potential in PCCs (Clarke *et al.* 1998, Kimura *et al.* 2014). Cilia incidence was reduced in PCCs/PGLs that had a Ki67 index of 3% or higher (*P* = 0.0159) compared to PCC/PGLs with a lower Ki67 index (*P* = 0.0159) (Fig. 1I). These data indicate that degree of cilia loss is linked to clinical parameters in PCC/PGLs.

### Dysregulation of cilia-mediated signaling pathways in PCCs

**Figure 2:**
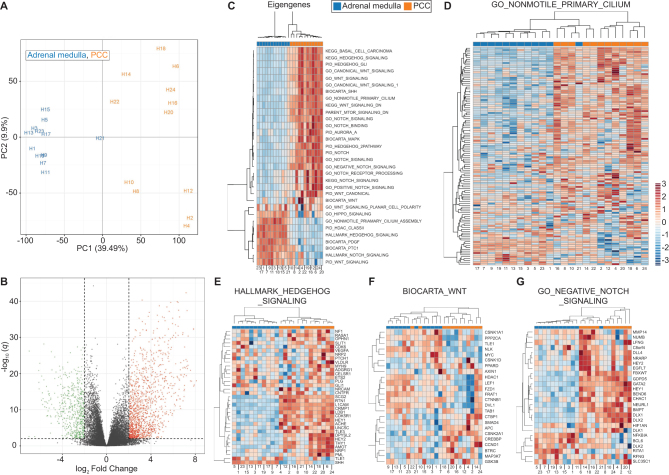
Changes in expression of cilia-linked genes in the transcriptomes of PCCs relative to adrenal medulla. (A) Principal component analysis (PCA) of RNA-seq expression data from 12 paired PCC and adjacent adrenal medulla tissue samples. (B) Volcano plot showing log_10_ FDR-adjusted *q* values versus log_2_ fold change between PCC and adjacent adrenal medulla. The vertical and horizontal dotted lines indicate 2× or −2× fold change and *q* = 0.01, respectively. (C) Heat map and hierarchical clustering depiction of all differentially expressed module eigengenes, from a collection of 32 gene sets known to be associated with cilia structure and cilia-mediated signaling, that are altered between PCCs and adjacent adrenal medulla. (D) Heat map and hierarchical clustering depiction of differentially expressed genes in the GO_NONMOTILE_PRIMARY_CILIUM pathway, comparing PCC and adjacent adrenal medulla samples. (E, F and G) Heat map and hierarchical clustering depictions of differentially expressed genes in three cilia-associated signaling pathways that are altered in PCCs relative to adjacent adrenal medulla: (E) HALLMARK_HEDGEHOG_SIGNALING; (F) BIOCARTA_WNT_PATHWAY; (G) GO_NEGATIVE_REGULATION_OF_NOTCH_SIGNALING_PATHWAY. Numbers shown at the bottom of the heat maps correspond to the sample IDs shown in the PCA (but are not prefixed with ‘H’).

We hypothesized that the reduced incidence and length of primary cilia in PCCs, relative to adrenal medulla, may result in alterations in cilia-mediated signaling. This was examined using RNA-Seq transcriptome analysis of 12 PCCs and adjacent adrenal medulla (Supplementary Table 1) to identify differentially expressed cilia-linked gene networks. We performed principal component analysis (PCA) on the filtered, normalized and transformed count matrix to explore the data’s latent structure (Fig. 2A). This revealed that principal component 1, which accounts for nearly 40% of all variation in the counts, separated the PCC samples from adjacent adrenal medulla. PCC samples were spread along principal component 2, which accounts for over 10% of data variance, indicating a heterogeneity in this group that is absent in adjacent adrenal medulla, where samples cluster together more closely.

Our unsupervised analysis suggested a strong transcriptomic signal differentiating tumor and adrenal medulla samples. To quantify this and identify relevant biomarkers, we conducted differential expression analysis using the DESeq2 software package (Love *et al.* 2014). We defined a gene as differentially expressed if its absolute log fold change ≥2 and its *q*-value was ≤0.01, imposing a false discovery rate of 1%. This strict threshold ensured high specificity. Overall, 1839 genes met these criteria, representing some 8% of the transcriptome after filtering (Fig. 2B).

To test if cilia function was altered in PCC relative to adjacent adrenal medulla, we curated a collection of 32 gene sets known to be associated with cilia structure and cilia-mediated signaling. We found considerable enrichment among these pathways (14 out of 32 at *q* ≤ 0.1). Eigengenes for all modules are depicted in Fig. 2C. Altered gene modules included those associated with cilia structure. For example, the GO_NONMOTILE_PRIMARY_CILIUM module showed altered expression in PCC tissue relative to adrenal medulla (*q* = 0.0519) (Fig. 2D), suggesting changes in gene expression may contribute to cilia loss in PCCs. We also observed changes in the Aurora-A Gene module (*q* = 0.2365), which is of interest as activation of Aurora-A pathway plays a role in regulation of cilia disassemble.

We also identified that gene modules associated with Hedgehog, Wnt and NOTCH signaling were altered between PCCs and adrenal medulla e.g. HALLMARK_HEDGEHOG_SIGNALING (*q* = 1.66 × 10^−7^), BIOCARTA_WNT_PATHWAY (*q* = 0.0519) and GO_NEGATIVE_REGULATION_OF_NOTCH_SIGNALING_PATHWAY (*q* = 1.58 × 10^−6^). Analyses of these gene modules revealed significant upregulation and downregulation of individual genes (absolute log fold change ≥2, *q* ≤ 0.01), while hierarchical clustering analyses separately grouped tumor and adrenal medulla samples in each of these pathways, with the exception of one medulla sample (H21) in the Hedgehog and Notch pathways, and two tumor samples (H10 and H22) in the Wnt pathway (Fig. 2D, E, F and G). These data are consistent with cilia-mediated signaling pathways being disrupted in PCC, but could also be explained by other potential mechanisms.

### Disruption of primary cilia function in the PCC-derived PC12 cell line promotes proliferation and alters expression of tumorigenesis-linked gene networks

**Figure 3:**
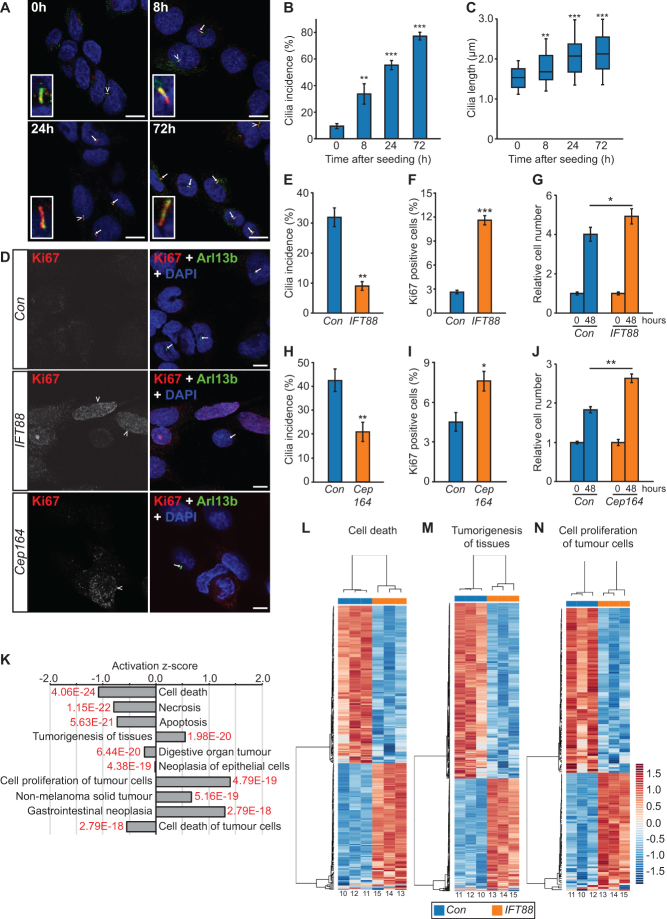
Loss of primary cilia in PC12 cells promotes proliferation and alters gene expression. (A) Confocal images of PC12 cells cultured in the absence of serum for between 0 and 72 h. Cells were immunolabeled with anti-acetylated α-tubulin (green) and anti-Arl13b (red) for detection of primary cilia. Nuclei were stained with DAPI (blue). Cilia are indicated by arrows, or arrowheads where they are also shown zoomed in the insets. Scale bars = 10µm. (B and C) Quantification of primary cilia incidence (B) and axonemal length (C) under conditions of serum starvation. (D) Confocal images of PC12 cells cultured for 48 h after transfection with siRNA targeting IFT88, Cep164, or non-targeting control siRNAs (Con). Cells were immunolabeled to detect cilia (Arl13b, green) and the proliferation marker Ki67 (red). Nuclei were stained with DAPI (blue). Cilia are indicated by arrows and Ki67 positive cells by arrowheads. Scale bars = 10 µm. (E, F, G and H) Quantification of primary cilia incidence (E), the percentage of Ki67 positive cells (F), and relative cell numbers (G), 48 h after transfection with siRNA targeting IFT88. (H, I and J) Quantification of primary cilia incidence (H), the percentage of Ki67 positive cells (I) and relative cell numbers (J), 48 h after transfection with siRNA targeting Cep164. Cilia and Ki67 scoring were performed in ten randomly selected fields for each experimental condition in three biological replicates. Mean axonemal length was quantified from at least 50 ciliated cells for each experimental condition. Cell counting was performed on six samples from three biological replicates. Error bars indicate 2× s.e.m. In box and whisker plots, the box represents median, upper and lower quartiles and the whiskers the 10th and 90th centiles. Statistical tests: ANOVA (B and C), *t*-test (E, F and G). *P < 0.05, **P < 0.01, ***P < 0.001. (K) Gene Ontology (GO) analysis of the transcriptome of PC12 cells transfected with siRNA targeting IFT88 or non-targeting control siRNAs, showing the top-ranking altered biological processes identified by Ingenuity Pathways Analysis. *q* values are depicted in red (*E* = 10 to the power of the following number). (L and M) Heat map and hierarchical clustering depictions of differentially expressed genes in altered pathways with the GO terms cell death (L), tumorigenesis of tissues (M) and cell proliferation of tumor cells (N). Numbers shown at the bottom of the heat maps correspond to sample IDs shown in Supplementary Fig. 3.

It is not fully resolved whether cilia loss is a driver or consequence of tumorigenesis. To address this question, in the context of PCC, we first established that PCC-derived cultured cell lines are able to form primary cilia. This was confirmed in the rat tumor-derived PC12 cell line, with cilia incidence and length increasing after serum starvation, such that 55.4 ± 5.98% of cells had a detectable cilium with a mean axonemal length of 2.17 ± 0.69 μm after 24 h (Fig. 3A, B, C and Supplementary Fig. 2A, B, C). Cilia were also present and responsive to serum starvation in two mouse PCC cell lines, MPC and MTT (Supplementary Fig. 2D, E and F). It should be noted that PC12 cells do not express the Myc dimerization partner MAX, while MPC and MTT lines were derived from the neurofibromatosis type 1 (NF1)-knockout mouse (Hopewell & Ziff 1995, Burnichon *et al.* 2012, Korpershoek *et al.* 2012).

We next disrupted cilia function in PC12 cells through siRNA-mediated knockdown of either the IFT88, a central component of the intraflagellar transport complex (Pazour *et al.* 2000), or Cep164, which plays a role in microtubule organization and/or maintenance for the formation of cilia (Graser *et al.* 2007). IFT88 knockdown was confirmed by immunoblot (Supplementary Fig. 3A and B), while knockdown of Cep164 was confirmed at the level of transcript (Supplementary Fig. 3C). Knockdown cells were then immunolabeled to detect cilia and stained with the proliferation marker Ki67 (Fig. 3D). Quantitative analysis confirmed, compared to control cells transfected with a non-targeting siRNA, that cilia incidence was reduced in both IFT88 (*P* = 0.02577) and Cep 164-knockdown cells (*P* = 0.0.00222) (Fig. 3E and H). Cilia length was also reduced in both instances (Supplementary Fig. 3D and E). Moreover, the percentage of Ki67-positive cells was increased after both IFT88 knockdown (*P* = 4.35 × 10^−12^) and Cep164 knockdown (*P* = 0.03937) (Fig. 3F and I). Increased proliferation of IFT88 and Cep164 knockdown PC12 cell, relative to controls, was further confirmed by quantification of cell numbers 48 h after siRNA transfection (Fig. 3G and J).

To further understand how disruption of cilia function impacts on cellular proliferation, we compared the transcriptomes of IFT88 knockdown and control cells (transfected with non-targeting siRNA) by RNA-Seq. Reads were pseudo-aligned (using the same pipeline as described for PCC and adrenal medulla) and PCA performed. PC1 separated IFT88 knockdown and control cells, accounting for over 30% of the variation in the counts (Supplementary Fig. 3F). We found 662 genes differentially expressed at *q* ≤ 0.01 (Supplementary Fig. 3G), representing some 6% of the transcriptome after filtering. Ingenuity pathways analysis was then used to identify statistically significant functions of the differentially regulated genes. This gene ontology (GO) analysis revealed that the top ten biological processes of these genes were related to cell death, cell proliferation and tumorigenesis. Moreover, activation *z*-scores suggested that cell death pathways were inhibited while proliferation and tumorigenesis pathways were induced (Fig. 3K). Hierarchical cluster analysis of gene modules described by the GO terms ‘cell death’, ‘tumorigenesis of tissues’, and ‘cell proliferation of tumor cells’ clearly separated IFT88-knockdown samples from controls (Fig. 3L, M and N). These data suggest that cilia loss promotes proliferation of PC12 cells.

### PC12 cells resorb primary cilia under hypoxic conditions

**Figure 4:**
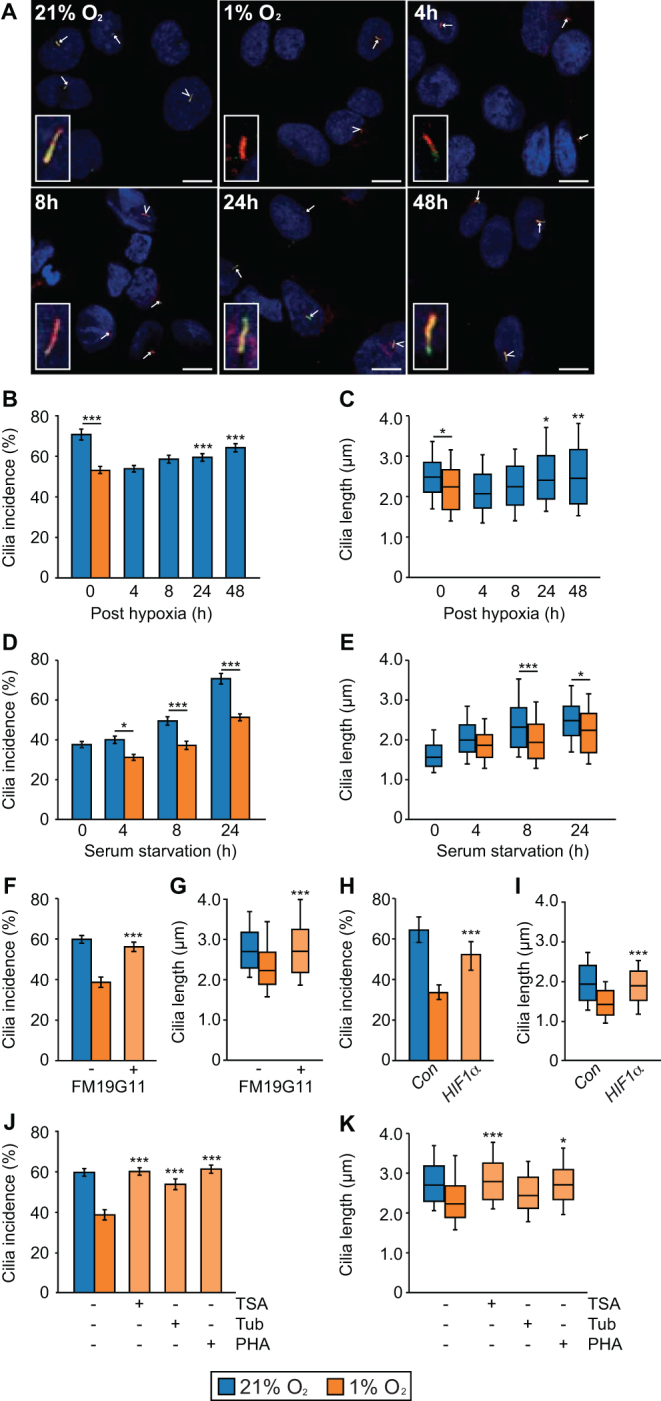
Primary cilia are lost from PC12 cells when oxygen levels are reduced. (A) Confocal images of PC12 cells cultured in 21% or 1% oxygen for 24 h, prior to return to 21% oxygen for 4, 8, 24 or 48 h before processing for the detection of primary cilia as in Fig. 3A. Scale bars = 10 µm. (B and C) Quantification of primary cilia incidence (B) and axonemal length (C) after 24 hours of culture in 21% and 1% oxygen and subsequent recovery in 21% oxygen. (D and E) Comparison of primary cilia incidence (D) and axonemal length (E) upon serum starvation after culture in 21% or 1% oxygen. (F and G) Quantification of primary cilia incidence (F) and axonemal length (G) after 24 h of culture in 1% oxygen in the presence of the HIFα inhibitor FM19G11 or vehicle only control. (H and I) Quantification of primary cilia incidence (H) and axonemal length (I) after 24 h of culture in 1% oxygen in cells transfected with non-targeting control siRNAs or siRNA targeting HIF1α. (J and K) Quantification of primary cilia incidence (J) and axonemal length (K) after 24 h of culture in 1% oxygen in the presence of the inhibitors trichostatin A (TSA), tubacin, PHA-680632 or vehicle only control. Cilia scoring was performed in ten randomly selected fields for each experimental condition in three biological replicates. Mean axonemal length was quantified from at least 50 ciliated cells for each experimental condition. Error bars indicate 2× s.e.m. Box and whisker plots are as in Fig. 3. Statistical tests: ANOVA. *P < 0.05, **P < 0.01, ***P < 0.001.

Primary cilia incidence was most reduced in tumors with germline mutations in *VHL* and *SDHx* (Fig. 1G). This suggested that hypoxic signaling may be a driver of cilia loss. To test this hypothesis, we exposed ciliated PC12 cells (grown in serum-free conditions for 24 h) to normal cell culture oxygen levels (21% O_2_) and hypoxic conditions (1% O_2_). Cells were then immunolabeled to detect cilia. Subsequent confocal imaging and quantitative analysis demonstrated that culture of ciliated PC12 cells in 1% O_2_ caused a reduction in cilia incidence (*P* = 3.75 × 10^−15^) and length (*P* = 0.0106) (Fig. 4A, B and C). This cilia resorption was shown to be transient, with PC12 cells able to reform primary cilia within 24 h of return to 21% O_2_ (Fig. 4B and C). We also looked at the effect of oxygen levels on ciliogenesis. Cilia formation, induced by culture in serum-free conditions, was compared in cells maintained under normoxic (21% O_2_) and hypoxic conditions (1% O_2_). Lowered oxygen levels again resulted in cells having a reduction in cilia incidence (*P* = 1.27 × 10^−5^) and length (*P* = 0.0075) (Fig. 4D and E). To further confirm cilia loss occurred in PCC-derived cell lines cultured under hypoxic conditions, we immunolabeled MPC and MTT cells to detect cilia. In MPC and MTT cell lines, primary cilia incidence (MPC *P* = 0.002; MTT *P* = 0.0351) and length (MPC *P* = 1.00 × 10^−15^; MTT *P* = 0.0001) was reduced after transfer to 1% O_2_ for 24 h (Supplementary Fig. 4).

To investigate if the loss of primary cilia under hypoxic conditions was dependent on HIF-mediated signaling, we added the HIFα inhibitor FM19G11 (Moreno-Manzano *et al.* 2010) to culture media prior to transfer of cells to 1% O_2_. Compared to vehicle-only-treated control cells, FM19G11 prevented hypoxia-induced cilia loss (*P* = 8.68 × 10^−5^ for incidence, *P* = 7.08 × 10^−6^ for length) (Fig. 4F, G and Supplementary Fig. 4D). We further tested a role for HIF1α signaling in hypoxia-induced cilia loss by targeting with siRNA. This showed that HIF1α knockdown was able to rescue cilia loss in PC12 cells cultured in 1% O_2_ (Fig. 4H, I and Supplementary Fig. 4E, F). To further understand the mechanism of hypoxia-induced cilia loss, we next tested for involvement of the Aurora-A kinase/histone deacetylase 6 (HDAC6) pathway. Activation of Aurora-A has been shown to cause phosphorylation of HDAC6, which deacetylates ciliary tubulin and destabilizes the axonemal microtubules (Pugacheva *et al.* 2007). Inhibition of Aurora-A, with the specific inhibitor PHA-680632, prevented cilia loss (*P* = 1.85 × 10^−7^) and shortening (*P* = 2.94 × 10^−6^) in cells exposed to 1% O_2_ (Fig. 4J, K and Supplementary Fig. 4D). Hypoxia-induced cilia loss was also inhibited by the mammalian class I and II HDAC inhibitor trichostatin A (TSA) (*P* = 3.22 × 10^−8^ for incidence, *P* = 3.29 × 10^−8^ for length) and the selective HDAC6 inhibitor tubacin (*P* = 1.11 × 10^−4^ for incidence, *P* = 0.0487 for length) (Fig. 4J, K and Supplementary Fig. 4D). These data suggest that reduced oxygen levels lead to cilia resorption in PC12 cells by a mechanism that includes HIF signaling and activation of the Aurora-A kinase/HDAC6 pathway.

In addition to degradation of HIF, pVHL stabilizes microtubules and plays a role in cilia maintenance. It is reported that loss of pVHL alone does not affect cilia structure but may sensitize cells to lose pre-established cilia (Thoma *et al.* 2007). pVHL has been shown to localize to the ciliary axoneme, and this was also the case in PC12 cells (Supplementary Fig. 4G). We thus investigated if activation of hypoxic signaling affected localization of pVHL by quantifying levels of the protein in the axoneme. Ciliary axonemes were detected by immunolabeling for acetylated tubulin and levels of pVHL that localized within the region of the cilia determined by analyses of fluorescent intensity. This showed that pVHL levels were reduced (*P* = 0.0234) in the cilium of cells maintained at 1% O_2_ relative to cells maintained at 21% O_2_ (Supplementary Fig. 4H). Thus, activation of hypoxic signaling may also destabilize cilia through a mechanism where pVHL is reduced in the ciliary axoneme.

### Pseudohypoxia in PC12 cells results in primary cilia loss and shortening

**Figure 5:**
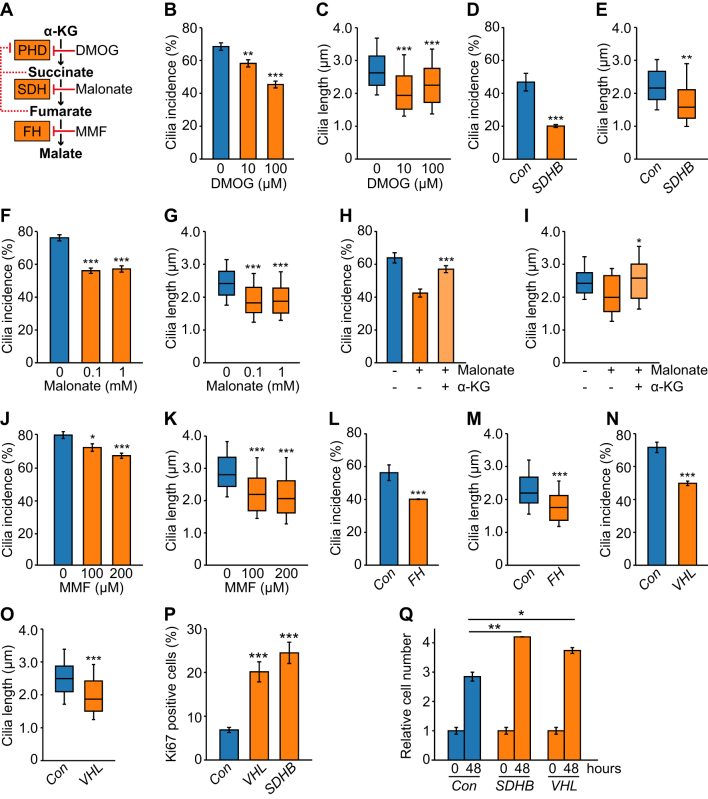
Inducers of pseudohypoxia cause primary cilia loss and shortening in PC12 cells. (A) Schematic showing PCC linked enzymes and inhibitors used to block their action. (B and C) Quantification of primary cilia incidence (B) and axonemal length (C) after 24 h of culture in the presence or absence of DMOG. (D and E) Quantification of primary cilia incidence (D) and axonemal length (E) 48 h after transfection with siRNAs targeting SDHB or non-targeting control siRNAs (*Con*). (F and G) Quantification of primary cilia incidence (F) and axonemal length (G) after 24 h of culture in the presence or absence of malonate. (H and I) Quantification of primary cilia incidence (H) and axonemal length (I) after 24 h of culture in the presence or absence of malonate (0.1 mM), with or without α-ketoglutarate (α-KG). (J and K) Quantification of primary cilia incidence (J) and axonemal length (K) after 24 h of culture in the presence or absence of monomethyl fumarate (MMF). (L, M, N and O) Quantification of primary cilia incidence (L and N) and axonemal length (M and O) 48 h after transfection with siRNAs targeting FH (L and M) or VHL (N and O) compared to non-targeting control siRNAs (*Con*). (P and Q) Quantification of the percentage of Ki67 positive cells (P) and of relative cell numbers (Q), 48 h after transfection with siRNAs targeting SDHB, VHL or control siRNAs. Cilia and Ki67 scoring was performed in ten randomly selected fields for each experimental condition in three biological replicates. Mean axonemal length was quantified from at least 50 ciliated cells for each experimental condition. Cell counting was performed on six samples from three biological replicates. Error bars indicate 2× s.e.m. Box and whisker plots are as in Fig. 3. Statistical tests: ANOVA (B, C, F, G, H, I, J, K, P, Q), *t*-test (D, E, L, M, N, O). *P < 0.05, **P < 0.01, ***P < 0.001.

Under normoxic conditions HIFα is hydroxylated at conserved proline residues by HIF prolyl-hydroxylases (HIF-PHDs). This leads to recognition of HIFα by VHL, facilitating their ubiquitination and subsequent proteasomal degradation. Thus, direct inactivation of either HIF-PHDs or VHL can result in persistence of HIFα and transcription of HIF target genes even in the presence of oxygen – pseudohypoxia. Moreover, succinate, which accumulates as a result of loss of SDH function, inhibits HIF-PHDs, again resulting in pseudohypoxia (Fig. 5A). To establish if pseudohypoxia impacted primary cilia, we firstly targeted HIF-PHs by treating PC12 cells with the inhibitor dimethyloxalylglycine, N-(methoxyoxoacetyl)-glycine methyl ester (DMOG). This resulted in reduced cilia incidence (*P* = 3.86 × 10^−11^) and length (*P* = 8.00 × 10^−14^) (Fig. 5B, C and Supplementary Fig. 5A). We next tested if drivers of the pseudohypoxic PCC/PGL phenotype resulted in cilia loss. For SDHB, siRNA-mediated knockdown (Supplementary Fig. 5B, C and D) again leads to a reduction in cilia incidence (*P* = 1.20 × 10^−8^) and length (*P* = 0.00124) (Fig. 5D and E). Cilia loss also occurred in the presence of malonate, which competes with succinate for active sites of SDH (Fig. 5F, G and Supplementary Fig. 5E). Malonate inhibition of SDH can be reversed by pharmacologically elevating intracellular α-ketoglutarate (MacKenzie *et al.* 2007). Consistent with this, we observed that addition of α-ketoglutarate to PC12 cells rescued the cilia loss phenotype observed in cells treated with malonate alone (Fig. 5H, I and Supplementary Fig. 5E). Similar to succinate, accumulation of fumarate, another citric acid cycle intermediate, inhibits HIF-PHDs (this is also linked to disease as germline mutations in FH cause PCC/PGL). We inhibited FH using the cell-permeable derivative of fumarate, monomethyl fumarate. This again resulted in the reduction in cilia incidence (*P* = 1.67 × 10^−5^) and length (*P* = 2.28 × 10^−17^) (Fig. 5J, K and Supplementary Fig. 5F). The same pattern of reduced cilia incidence (*P* = 4.11 × 10^−6^) and length (*P* = 5.75 × 10^−19^) was observed when siRNA-mediated knockdown of FH (Supplementary Fig. 5G, H and I) was performed (Fig. 5L and M).

Finally, we investigated the effect of siRNA-mediated knockdown of VHL (Supplementary Fig. 5J, K and L) on primary cilia. Quantification of cilia incidence and length showed that VHL knockdown resulted in fewer cells exhibiting a cilium (*P* = 6.93 × 10^−13^) and that mean cilia length was decreased (*P* = 8.12 × 10^−14^) (Fig. 5N and O). In summary, these data show cilia loss was induced by a number of different conditions that impair HIFα degradation, including those that lead to accumulation of oncometabolites. Importantly, knockdown of *Sdhb* and *Vhl* also resulted in increased Ki67 labeling and cell number, relative to control cells transfected with a non-targeting siRNA (Fig. 5P and Q). This is consistent with pseudohypoxia-induced cilia loss correlating with increased cellular proliferation.

### Inhibition of both the Aurora-A/HDAC6 cilia resorption pathways and of hypoxic signaling prevents cilia loss in SDHB and VHL-knockdown cells

**Figure 6:**
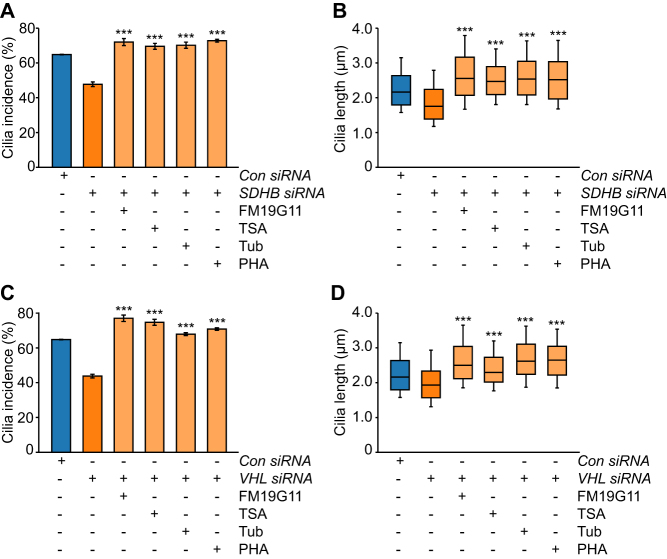
Inhibition of cilia resorption and hypoxic signaling prevents cilia loss caused by knockdown of SDHB and VHL. (A and B) Quantification of primary cilia incidence (A) and axonemal length (B) 48 h after transfection with siRNAs targeting SDHB in the presence or absence of the inhibitors FM19G11, TSA, tubacin (Tub) and PHA-680632 (PHA), or vehicle only controls. Cells transfected with non-targeting control siRNAs (*Con*) were treated with the same inhibitors. (C and D) Quantification of primary cilia incidence (C) and axonemal length (D) 48 h after transfection with siRNAs targeting VHL in the presence or absence of the inhibitors used in Fig. 6A and B. Cilia scoring was performed in ten randomly selected fields for each experimental condition in three biological replicates. Mean axonemal length was quantified from at least 50 ciliated cells for each experimental condition. Error bars indicate 2× s.e.m. Box and whisker plots are as in Fig. 3. Statistical tests: ANOVA. *P < 0.05, **P < 0.01, ***P < 0.001.

To understand why cilia incidence and length was reduced upon depletion of *SDHB* or *VHL*, we tested whether inhibition of the Aurora-A/HDAC6 pathway prevented cilia loss. PC12 cells were transfected with siRNAs targeting *Sdhb* or *Vhl* and then cultured in media containing PHA-680632, TSA, tubacin or vehicle only as a control. Forty-eight hours after transfection, cells were fixed and cilia were immunolabeled for confocal microscopy. Quantification of cilia incidence and length showed that treatment with the Aurora-A inhibitor PHA-680632 and the HDAC inhibitors TSA and tubacin reduced cilia loss and shortening in response to pVHL and SDHB (Fig. 6A, B, C, D and Supplementary Fig. 6) knockdown. Inhibition of HIF signaling with FM19G11 also reduced cilia loss in both *Sdhb*- and *Vhl*-depleted cells (Fig. 6A, B, C, D and Supplementary Fig. 6). Together, these data indicate that the Aurora-A/HDAC6 pathway is a modulator of cilia loss in PC12 cells depleted for SDHB or pVHL.

## Discussion

Data presented here are the first to show that primary cilia are lost from PCCs compared to normal adjacent adrenal medulla. This corresponds with observations that primary cilia structure and function is disrupted in a broad range of cancers (O’Toole & Chapple 2016). These include breast, prostate, renal, pancreatic, melanoma, cholangiocarcinoma, glioblastoma, chondrosarcoma and colon cancer (Moser *et al.* 2009, Schraml *et al.* 2009, Seeley *et al.* 2009, Yuan *et al.* 2010, Kim *et al.* 2011, Gradilone *et al.* 2013, Hassounah *et al.* 2013, Ho *et al.* 2013, Rocha *et al.* 2014). In PCCs, the degree of cilia loss was more pronounced in tumors from patients with germline mutations in pseudohypoxia-linked genes *VHL* and *SDHB*. For pVHL, this may be partly explained by its reported non-canonical function in ciliogenesis, by orienting growth of microtubules toward the cell periphery (Schermer *et al.* 2006). Cilia frequency is also reduced relative to neighboring tissue in ccRCC. The *VHL* gene is inactivated in the majority (87%) of sporadic clear-cell RCCs (Moore *et al.* 2011), with ccRCCs also occurring as part of von Hippel–Lindau disease (Gossage *et al.* 2015, Crespigio *et al.* 2017). Contrasting ccRCC, *VHL* inactivation is a much less common feature of sporadic PCCs (Dannenberg *et al.* 2003, Burnichon *et al.* 2011). In the context of our data, this suggests that although disruption of a cilia-specific function of VHL may contribute to loss of cilia in PCC, it is not the main mechanism responsible for cilia loss.

Using the PCC-derived PC12 cell line, we found that siRNA-mediated depletion of SDHB, FH and VHL, all resulted in reduction of cilia frequency. We also observed that treatment of cells with drugs that inhibit HIF-PHs, SDH and FH, leading to accumulation of oncometabolites for SDH and FH, caused cilia loss. Culture of PC12 cells in conditions of reduced oxygen similarly reduced the incidence of cilia, although it should be noted the change in oxygen concentration from 21% (standard for cell culture) to 1% is greater than will to occur *in vivo*, where physiological levels of oxygen range from 2 to 9% (Tiede *et al.* 2011). Together, these data implicate pseudohypoxic/hypoxic signaling as a regulator of cilia dynamics. This is further supported by the finding that inhibition of HIF signaling reduced cilia loss in response to hypoxia and inducers of pseudohypoxia and is consistent with studies that show axoneme length is influenced by hypoxia-inducible mechanisms (Proulx-Bonneau & Annabi 2011, Wann *et al.* 2013). Hypoxia is not just a driver of PCC/PGL formation (Rodriguez-Cuevas *et al.* 1986, Opotowsky *et al.* 2015), but is also a salient feature of many solid tumors, and may therefore modulate cilia presence in cancers more generally.

There are a number of potential pathways through which HIF signaling could influence ciliogenesis and resorption. These include that stabilization of HIF promotes transcription of Aurora-A kinase, which functions in regulation of ciliary resorption with HDAC6 (Pugacheva *et al.* 2007, Xu *et al.* 2010). Inhibition of the Aurora-A/HDAC6 pathway in PC12 cells prevented cilia loss in response to hypoxia and induction of pseudohypoxia. Collectively, these findings suggest that the reduction in cilia frequency in pseudohypoxic PCC is likely to be mediated by both HIF signaling and the Aurora-A/HDAC6 cilia resorption pathway, although involvement of other regulators of cilia dynamics is also possible.

Transcriptome analyses identified altered expression of gene modules associated with cilia-mediated signaling in PCCs relative to adjacent adrenal medulla. Altered cilia-mediated signaling pathways included Hh, WNT and Notch signaling. The role of cilia in the regulation of cancer-linked signaling pathways is complex and context dependent (Oh & Katsanis 2013). For example, WNT signaling, which is generally considered to be attenuated by the presence of a cilium, can be decreased in cells with shortened cilia yet activated by ablation of cilia (Lancaster *et al.* 2011, Oh & Katsanis 2013), while for Hh signaling, the cilium activates the pathway in the presence of the sonic hedgehog ligand (SHH) and restrains signaling when SHH is absent (Wong *et al.* 2009, Hassounah *et al.* 2012). There is also crosstalk between cilia-mediated signaling pathways, such as Notch signaling modulating SHH signaling, by regulating the ciliary localization of the Hh signal transduction proteins patched and smoothened (Kong *et al.* 2015). This complexity makes it difficult to interpret how alterations in cilia incidence and length may impact on specific pathways. Nevertheless, hierarchical clustering analyses separately grouped tumor and adrenal medulla samples based on changes of gene expression in multiple cilia-linked signaling pathways. This is consistent with loss of cilia correlating with dysregulation of cilia-mediated signaling in PCCs. Disruption of WNT signaling is particularly relevant to PCC/PGLs, with WNT-altered tumors classified as one of four molecularly defined PCC/PGL subtypes (Fishbein *et al.* 2017).

In addition to modulating signaling pathways that are dysregulated in tumorigenesis and cancer, the presence of a primary cilium may act as a checkpoint for cell division. We observed that disruption of cilia structure and function, by knockdown of *Ift88* or *Cep164*, correlated with increased cellular proliferation of PC12 cells. This was accompanied by changes in gene expression that inhibited cell death pathways, while activating cell proliferation and tumor-linked pathways. Together, our data are concordant with cilia acting as a checkpoint for cell division in PC12 cells and suggest cilia loss promotes proliferation and perhaps tumorigenesis in PCC/PGL. Interestingly, we also observed that in PCC tissue, there was a correlation between degree of cilia loss and Ki67 staining implying *in vivo* relevance of our findings in PC12 cells.

**Figure 7:**
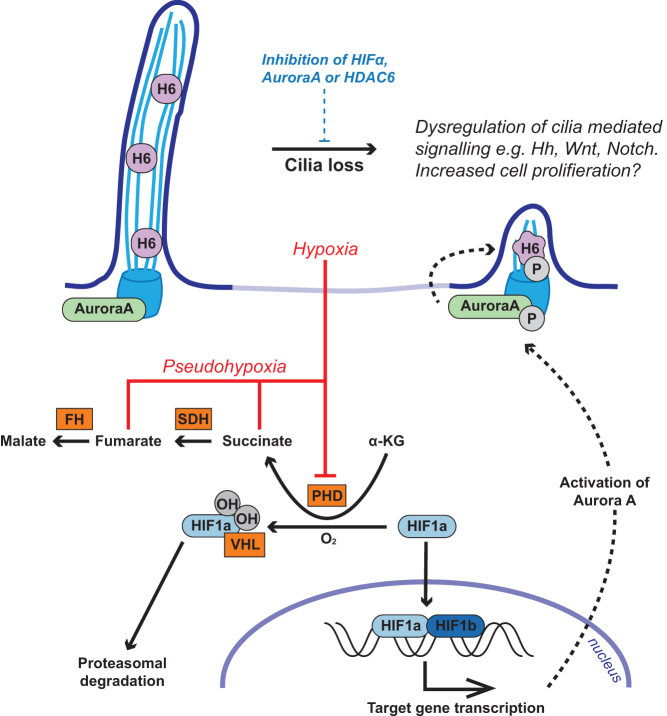
Model illustrating the potential pathway from pseudohypoxia-induced cilia loss to increased cell proliferation and dysregulation of tumorigenesis relevant cilia-mediated signaling pathways in PCCs. Proteins where germline mutations in the gene predispose to PCC are in orange boxes. Aurora-A, Aurora-A kinase; FH, fumarate hydratase; H6, HDAC6; HIF, hypoxia-inducible factor; OH, hydroxyl group; P, phosphate group; PH, prolyl-hydroxylases; SDH, succinate dehydrogenase; VHL, von Hippel–Lindau protein.

In summary, we propose that in PCC/PGLs oncometabolite-induced pseudohypoxia drives cilia loss through activation of the Aurora-A kinase/HDAC6 cilia resorption pathway. In PCCs, this cilia loss causes dysregulation of cilia-mediated signaling pathways including SHH, WNT and Notch signaling and is also likely to promote increased cellular proliferation (Fig. 7). Hypoxia-induced cilia resorption may be a feature of cancers more generally and represents a potential target to slow tumor progression.

## Supplementary Material

Supporting Information

Supporting Table 1

Supporting Table 2

Supporting Figure 1

Supporting Figure 2

Supporting Figure 3

Supporting Figure 4

Supporting Figure 5

Supporting Figure 6

## Declaration of interest

The authors declare that there is no conflict of interest that could be perceived as prejudicing the impartiality of the research reported.

## Funding

This work was funded by a Barts and the London Charity Clinical Research Training Fellowships awarded to Samuel M O’Toole (grant number MRD0191) and by a project grant from the UK Medical Research Council (MRC) (grant number MR/L002876/1). Dr Tyson V Sharp is funded by grants from the UK Biotechnology and Biological Sciences Research Council (grant number BB/M0020611) and MRC (grant number MR/N009185/1). The LSM880 confocal used in these studies was purchased through a Barts and the London Charity award (grant number MGU0293). The RNA-Seq analyses were in part funded by a grant from The Medical College of Saint Bartholomew’s Hospital Trust and were performed at the Barts and the London Genome Centre. This work also forms part of the research themes contributing to the translational research portfolio of Barts and the London Cardiovascular Biomedical Research Centre, which is supported and funded by the National Institute of Health Research. This project was enabled through access to the MRC eMedLab Medical Bioinformatics infrastructure, supported by the MRC (grant number MR/L016311/1).

## Author contribution statement

S M O, P J K, M M K, T V S, U S, W M D and J P C conceived and planned the experiments. S M O, L E L R, T V N, T Y B and C L T carried out the experiments. S M O, D S W, M R B and J P C analyzed and interpreted the data. S M O and J P C wrote the draft manuscript. All authors provided critical feedback, which shaped the research, analysis and the manuscript.
