# Supplementary material for: Oncometabolite induced primary cilia loss in pheochromocytoma
Source: Endocr Relat Cancer. 2018 Sep 5;26(1):165–80. doi: 10.1530/ERC-18-0134 (PMC6215910; doi:10.1530/ERC-18-0134)
Supplement: Supporting Information [file erc-26-165-t001.pdf]

Supplemental Information

Material and Methods

Patient recruitment and ethical approval

Consecutive patients who were due to undergo surgery for PCC or PGL (diagnosis based on pre-operative imaging and biochemistry with subsequent pathological confirmation) at St Bartholomew's Hospital, University College London Hospital and the National Hospital for Neurology and Neurosurgery (all London, UK) were approached for involvement in this study. All participants gave written informed consent for the study, which was approved by Cambridge East Research Ethics Committee (REC: 06/Q0104/133). Clinical data was obtained from participants' medical records.

Cell Culture and experimental treatments

PC-12 were cultured in F-12K medium supplemented with 15% horse serum (HS), 2.5% heat-inactivated fetal bovine serum (FBS) and 50U/ml penicillin and 50 μg ml^{-1} streptomycin (final concentration in media, 1%). MPC and MTT cells were cultured in Dulbecco's Minimum Eagle Medium (DMEM) supplemented with 10% FBS and 50U/ml penicillin and 50 μg ml^{-1} streptomycin (final concentration in media, 1%). All cells were kept in a constant humidified atmosphere of 5% CO_2 at 37°C and were confirmed free of mycoplasma contamination by qPCR. Cells were grown either in atmospheric oxygen (21%) or in 1% O_2 for hypoxic experiments using a hypoxic workstation, where nitrogen was the balancing gas. Cell culture reagents were from Life Technologies (Paisley, UK). All drugs were obtained from Sigma (UK) unless otherwise stated. Cells were treated for the duration of cilia formation (48 hours for si-RNA treatments, 24 hours for others). Unless otherwise stated, final concentrations of drugs used were as follows: FM19G11 500 nM, PHA-680632 100 nM (Selleck Chemicals), Trichostatin A 10 nM, Tubacin 500 nM, DMOG 100 uM, malonate 0.1 mM, α-ketoglutarate 0.1 mM, MMF 400 uM. Vehicle only (water for malonate, α-ketoglutarate and MMF; DMSO for all others) controls were also performed.

Immunohistochemistry and Immunocytochemistry

For immunofluorescent detection of cilia in tissue samples, 20um thick free-floating cryosections were utilised. For cell culture specimens, cells were grown on glass coverslips and fixed with 4% formaldehyde for 10 minutes prior to immunofluorescent detection of cilia. Both tissue sections and cell monolayers were subsequently permeabilized for 5 minutes with 0.25% Triton-X 100 in phosphate buffered saline (PBS) prior to incubation in a blocking solution of 3% bovine serum albumin and 5% goat serum in PBS for 1 hour. Samples were then incubated with primary antibodies for 2 hours at room temperature or overnight at 4°C in blocking solution, prior to washing and incubation with fluorescently labelled secondary antibodies (1:1000, Alexa Fluor 488-

conjugated goat anti-rabbit or anti-mouse, Alexa Fluor 543-conjugated goat anti-mouse or anti-rabbit, Molecular Probes). Nuclei were counterstained with DAPI (4',6-diamidino-2-phenylindole) and coverslips mounted for microscopy using Dako Fluorescence Mounting Media (Agilent). Primary antibodies were used at the following titres: 1:2000 for mouse monoclonal anti-acetylated α-tubulin (clone 611 B-11, Sigma), 1:500 for rabbit polyclonal anti-Arl13b (17711-1-AP, Proteintech), 1:500 rabbit polyclonal anti-VHL (sc-5575, Santa Cruz, USA) and 1:600 rabbit monoclonal anti-Ki67 (EPR3610, Abcam).

Immunoblotting

Proteins were resolved using precast 4–12% gradient NuPAGE Bis-Tris gels (Life Technologies) and transferred to nitrocellulose membranes (Whatman), blocked in 5% (w/v) non-fat milk powder before probing with the specified antibodies. Images of the immunoblots were visualised and quantified using the LiCor Odyssey. Primary antibodies were used at the following titres; 1:10,000 for mouse monoclonal anti-β-actin (AC-15, Abcam), 1:5,000 for rabbit polyclonal anti-GAPDH (ab9485, Abcam), 1:1000 for rabbit polyclonal anti-IFT88 (13967-1-AP, Proteintech), 1:1000 for mouse monoclonal anti-SDHB (21A11AE7, Abcam), 1:1000 for rabbit polyclonal anti-VHL (sc5575, Santa Cruz) and 1:1000 for rabbit monoclonal anti-FH (EPR11648, Abcam). Immunoreactive products were visualized and quantified, after labelling with species-specific infrared secondary antibodies (IRDye®680LT Goat anti-Mouse 1:5000 LI-COR , IRDye®800CW Goat anti-Rabbit 1:5000 LI-COR Biosciences), using the Odyssey imaging system (LI-COR). Apparent molecular masses were estimated using the Novex Sharp Pre-stained Protein Standard (Life Technologies).

RNA extraction and sequencing

Total RNA was isolated from tissue samples and PC12 cells using the RNeasy Mini kit (Qiagen, UK). Total RNA samples were quantified using a Nanodrop (Thermo Fisher Scientific) and the integrity was checked using the Bioanalyser (Agilent Technologies). 100 ng of RNA with RIN >7 was used as input in the KAPA Stranded RNA-Seq with RiboErase (Roche) as per manufacturer's instructions to generate libraries. Libraries were quantified by spectrophotometry using a Nanodrop (ThermoFischer) and library fragment size estimated by Tapestation (Agilent Technologies). Three equal library pools were made and 12pM loaded onto three Illumina NextSeq 500 flow cells and sequenced to 75bp Paired End, as per manufacturer's instructions.

RNA sequence data and pathway analyses

Following RNA-sequencing of all samples, reads were pseudo-aligned using kallisto (Bray et al., 2016). Transcript-level counts were aggregated to gene-level with the tximport package (Soneson et al., 2015). Prior to exploratory data analysis (EDA), we filtered low count genes based on

previous guidelines (Robinson et al., 2010); normalised libraries using the relative log expression method (Anders and Huber, 2010); and applied the $\log_2$-counts per million transformation, which stabilises variance for unsupervised analysis. This matrix was also used to test for pathway enrichment (see below), but not for differential expression. Each gene was tested for differential expression between adrenal and tumor tissue using tools from the DESeq2 analysis pipeline. We filtered genes and estimated *q*-values using the package's independent hypothesis weighting method (Ignatiadis et al., 2016).

To detect pathway enrichment in gene sets associated with cilia regulation, we implemented a modified version of the QuSAGE algorithm (Turner et al., 2015; Yaari et al., 2013), designed for modelling RNA-seq data. This method tests for overexpression in the residual matrix among user-supplied gene sets, following the same filtering, normalisation, and transformation steps described above for EDA. Pathway *q*-values were calculated using Storey's method (Storey and Tibshirani, 2003). Gene sets were summarised to vectors of length 24 (our study's sample size) by taking the first principal component of each, i.e. by calculating the module eigengene. Cilia structure and cilia-mediated signalling gene sets were curated from the Broad Institute's Molecular Signatures Database v6.0 (https://www.software.broadinstitute.org/gsea/msigdb). This collection included pathways from the Kyoto Encyclopedia of Genes and Genomes (http://www.genome.jp/kegg/) and the Gene Ontology Consortium (http://www.geneontology.org/). Data from transcriptome experiments has been deposited on ArrayExpress.

Statistical analyses

All data are expressed as means SEM unless otherwise stated. For cilia length box and whisker plots, the box represents the interquartile range, with the centre line the median and the whiskers represent the 10th and 90th centiles. Statistical significances were determined using two-tailed Student's t-test or one-way ANOVA as appropriate.
