# Supplementary material for: Oncometabolite induced primary cilia loss in pheochromocytoma
Source: Endocr Relat Cancer. 2018 Sep 5;26(1):165–80. doi: 10.1530/ERC-18-0134 (PMC6215910; doi:10.1530/ERC-18-0134)
Supplement: Supporting Table 1 [file erc-26-165-t002.pdf]

Supplemental Table 1: Clinical details – paired samples

Sample ID (RNAseq ID)	Sex	Age at operation	Pheo/PGL	Location	Mode of diagnosis	Syndromic	Genetics screening	Gene mutation	Max size/mm	NMN	MN	3MOT
1[A] (H1-2)	M	49	Pheo	Right	Symptomatic	No	No		36	-	+	-
2[A] (H3-4)	F	71	Pheo	Right	Incidental	No	No		32	+	+	-
3[A] (H5-6)	M	12	Pheo	Left	Symptomatic	No	Yes	Negative	58	+++	-	+
4[A, B] (H7-10)	M	37	Pheo	Left	Screening	Yes	Yes	MEN2	14	-	+	-
5[A] (H11-12)	F	31	Pheo	Left	Symptomatic	No	Yes	Negative	49	+++	-	+
6[A] (H13-14)	M	18	Pheo	Left	Screening	Yes	Yes	VHL	32	++	-	+
7[A] (H15-16)	M	69	Pheo	Right	Incidental	No	No		80	+++	+++	+
8[A] (H17-18)	M	39	Pheo	Left	Incidental	No	Yes	SDHB	48	+	-	-
9[A] (H19-20)	F	34	Pheo	Right	Symptomatic	No	Yes	Negative	76	+++	-	+
10[A] (H21-22)	M	64	Pheo	Left	Incidental	No	Yes	Negative	33	+	-	-
11[A, C] (H23-24)	M	14	Pheo	Right	Screening	Yes	Yes	VHL	14	+	-	+
12		14		Left	Screening	Yes	Yes	VHL	8	+	-	-
13	F	27	Pheo	Right	Symptomatic	No	Yes	Negative	54	+++	-	+
14	F	66	Pheo	Right	Incidental	No	No		47	+	-	-
15	F	69	Pheo	Right	Incidental	No	No		57	+	-	-
16	F	13	Pheo	Left	Symptomatic	Yes	Yes	VHL	23	+++	-	-
17		13		Right	Symptomatic	Yes	Yes	VHL	49	+++	-	-
18	F	35	Pheo	Right	Incidental	No	Yes	Negative	73	++	+++	+
19	F	78	Pheo	Left	Incidental	No	No		45	+	++	-
20	M	63	Pheo	Right	Incidental	No	No		35	+	++	-
21	F	52	Pheo	Right	Symptomatic	No	No		30	+	+	-
22	M	43	Pheo	Right	Incidental	No	No		56	++	+++	-
23	M	46	Pheo	Right	Symptomatic	No	Yes	Negative	75	++	-	+
24	F	74	Pheo	Left	Incidental	No	No		76	++	+++	++
25	F	50	Pheo	Right	Incidental	No	Yes	Negative	80	++	+++	+
26	F	60	Pheo	Right	Incidental	No	No		87	+++	+++	+
27	M	56	Pheo	Right	Incidental	No	No		55	+	++	-

Supplemental Table 1: Clinical details of 27 paired samples of tumour and adjacent adrenal samples from 25 individuals. F female, M male. Pheo pheochromocytoma, PGL paraganglioma. Mode of diagnosis – symptomatic = diagnosis due to symptoms or signs of catecholamine excess leading to diagnosis; incidental = diagnosis due to investigation for another unrelated condition; screening = diagnosis during a screening programme in individuals with known pheo/PGL predisposition. SDHB succinate dehydrogenase B, VHL von Hippel-Lindau, MEN2 Multiple Endocrine Neoplasia Type 2. NMN normetanephrine, MN metanephrine, 3MOT 3-methoxytramine; - not elevated, + elevated 1-5x upper limit of normal (ULN), ++ elevated 5-10x ULN, +++ elevated >10x ULN.

[A] Samples included in the RNAseq analysis (RNAseq sample ID in brackets; odd number adrenal medulla, even number pheochromocytoma)

[B] Patient 4 had synchronous bilateral disease which was included in RNAseq analysis but immunochemistry was only available in the left pheochromocytoma

[C] Patient 11 had metachronous bilateral disease and RNAseq was performed on one set of paired samples only
