# Supplementary material for: Oncometabolite induced primary cilia loss in pheochromocytoma
Source: Endocr Relat Cancer. 2018 Sep 5;26(1):165–80. doi: 10.1530/ERC-18-0134 (PMC6215910; doi:10.1530/ERC-18-0134)
Supplement: Supporting Table 2 [file erc-26-165-t003.pdf]

Supplemental Table 2: Clinical details – unpaired samples

ID	Sex	Age at operation	Pheo/PGL	Location	Mode of diagnosis	Syndromic	Genetics screening	Gene mutation	Max size/mm	NMN	MN	3MOT
28	M	43	PGL	H&N	Symptomatic	Yes	Yes	SDHB	20	-	-	-
29	F	44	PGL	H&N	Symptomatic	Yes	Yes	SDHB	18	-	-	-
30	F	68	PGL	Bladder	Symptomatic	Yes	Yes	SDHB	45	+	-	+++
31<br>32<br>33	F	49	PGL	Thoracic	Symptomatic	Yes<br>Yes<br>Yes	Yes<br>Yes<br>Yes	SDHA<br>SDHA<br>SDHA	58	+	-	++
34<br>35<br>36	M	15	Pheo	Right	Screening<br>Screening<br>Screening	Yes<br>Yes<br>Yes	Yes<br>Yes<br>Yes	VHL<br>VHL<br>VHL	35	+	-	-
37	F	43	Pheo	Right	Incidental	No	Yes	Negative	60	+++	+	-
38	F	53	PGL	Abdo	Incidental	No	No	[redacted]	56	+	-	-
39	M	45	Pheo	Right	Symptomatic	No	No	[redacted]	110	+++	+++	+
40	M	66	Pheo	Right	Incidental	No	Yes	Negative	120	+++	-	++
41	F	50	PGL	Abdo	Incidental	No	No	[redacted]	52	++	-	-
42	F	36	PGL	Abdo	Symptomatic	Yes	Yes	VHL	25	+	-	-
43	M	65	PGL	Abdo	Incidental	No	No	[redacted]	45	+	-	-
44	M	39	PGL	H&N	Symptomatic	No	Yes	Negative	20	-	-	-
45	M	37	Pheo	Right	Screening	Yes	Yes	MEN2	13	-	+	-
46<br>47	F	40	PGL	Abdo	Incidental	Yes	Yes	SDHB	39<br>33	+	-	-

Supplemental Table 2: Clinical details of 20 tumour samples from 15 individuals.

F female, M male. Pheo pheochromocytoma, PGL paraganglioma. H&N head and neck, abdo abdominal. Mode of diagnosis – symptomatic = diagnosis due to symptoms or signs of catecholamine excess leading to diagnosis; incidental = diagnosis due to investigation for another unrelated condition; screening = diagnosis during a screening programme in individuals with known pheo/PGL predisposition. SDH succinate dehydrogenase, VHL von Hippel-Lindau, MEN2 Multiple Endocrine Neoplasia Type 2. NMN normetanephrine, MN metanephrine, 3MOT 3-methoxytramine; - not elevated, + elevated 1-5x ULN, ++ elevated 5-10x ULN, +++ elevated >10x ULN.
