# Supplementary material for: Oncometabolite induced primary cilia loss in pheochromocytoma
Source: Endocr Relat Cancer. 2018 Sep 5;26(1):165–80. doi: 10.1530/ERC-18-0134 (PMC6215910; doi:10.1530/ERC-18-0134)
Supplement: Supporting Figure 1 [file erc-26-165-s001.pdf]

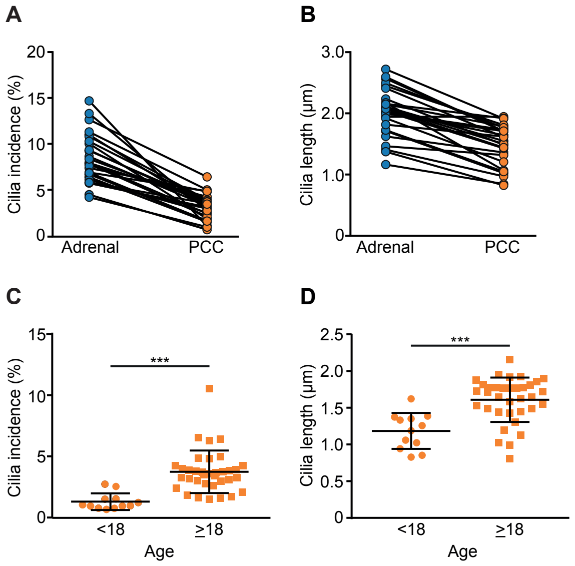

Figure S1. Primary cilia incidence and length is reduced in PCCs relative to adjacent adrenal medulla and this cilia loss correlates with clinical disease parameters. (A) Quantification of the percentage of cells that exhibited a detectable primary cilium in 27 paired PCC and adjacent adrenal medulla tissue samples. **(B)** Quantification of axoneme length from cells that had a primary cilium in PCC and adjacent adrenal medulla. Paired samples are linked by solid lines. **(C)** Comparison of cilia incidence in PCC from patients who were under or over 18 years of age at the time of surgery. **(D)** Comparison of axoneme length from cells that had a primary cilium in PCC from patients who were under or over 18 years of age at the time of surgery. The number of cilia and nuclei were counted in 15 randomly selected fields for each sample. Mean axonemal length was quantified from at least 50 ciliated cells for each sample. Error bars indicate SD. Statistical tests: t-test (C-D). * $P<0.05$, ** $P<0.01$, *** $P<0.001$.
