# Supplementary material for: Oncometabolite induced primary cilia loss in pheochromocytoma
Source: Endocr Relat Cancer. 2018 Sep 5;26(1):165–80. doi: 10.1530/ERC-18-0134 (PMC6215910; doi:10.1530/ERC-18-0134)
Supplement: Supporting Figure 2 [file erc-26-165-s002.pdf]

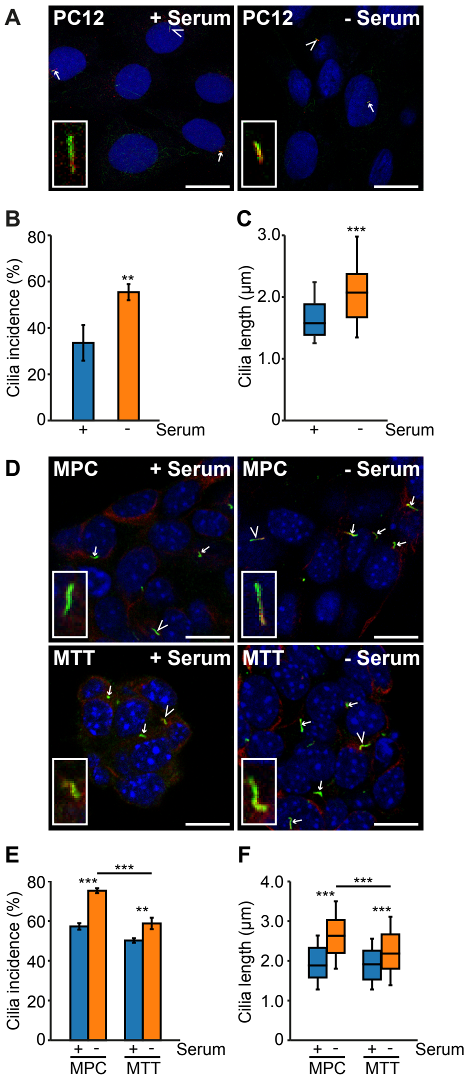

Figure S2. PCC derived cell lines express primary cilia and siRNA-mediated knockdown of IFT88 in PC12 cell. (A) Confocal images of rat PC12 cells cultured in the presence or absence of serum for 24 hours. Cells were immunolabelled for detection of primary cilia as in Figure 3. **(B, C)** Quantification of primary cilia incidence **(B)** and axonemal length **(C)** in PC12 cells after 24 hours of serum starvation. **(D)** Confocal images of mouse MPC and MTT cells cultured in the presence or absence of serum for 24 hours. Cells were immunolabelled for detection of primary cilia as in Figure 3. Cilia are indicated by arrows, or arrowheads where they are also shown zoomed in the insets. **(E, F)** Quantification of primary cilia incidence **(E)** and axonemal length **(F)** in MPC and MTT cells after 24 hours of serum starvation. Cilia scoring was performed in 10 randomly selected fields for each experimental condition in three biological replicates. Mean axonemal length was quantified from at least 50 ciliated cells for each experimental condition. Cilia are indicated by arrows, or arrowheads where they are also shown zoomed in the insets. Scale bars = 10µm. Error bars indicate 2x SEM. In box and whisker plots, the box represents median, upper and lower quartiles and the whiskers the 10th and 90th centiles. Statistical tests: ANOVA (B-C). * P<0.05, **P<0.01, ***P<0.001.
