# Supplementary material for: Oncometabolite induced primary cilia loss in pheochromocytoma
Source: Endocr Relat Cancer. 2018 Sep 5;26(1):165–80. doi: 10.1530/ERC-18-0134 (PMC6215910; doi:10.1530/ERC-18-0134)
Supplement: Supporting Figure 3 [file erc-26-165-s003.pdf]

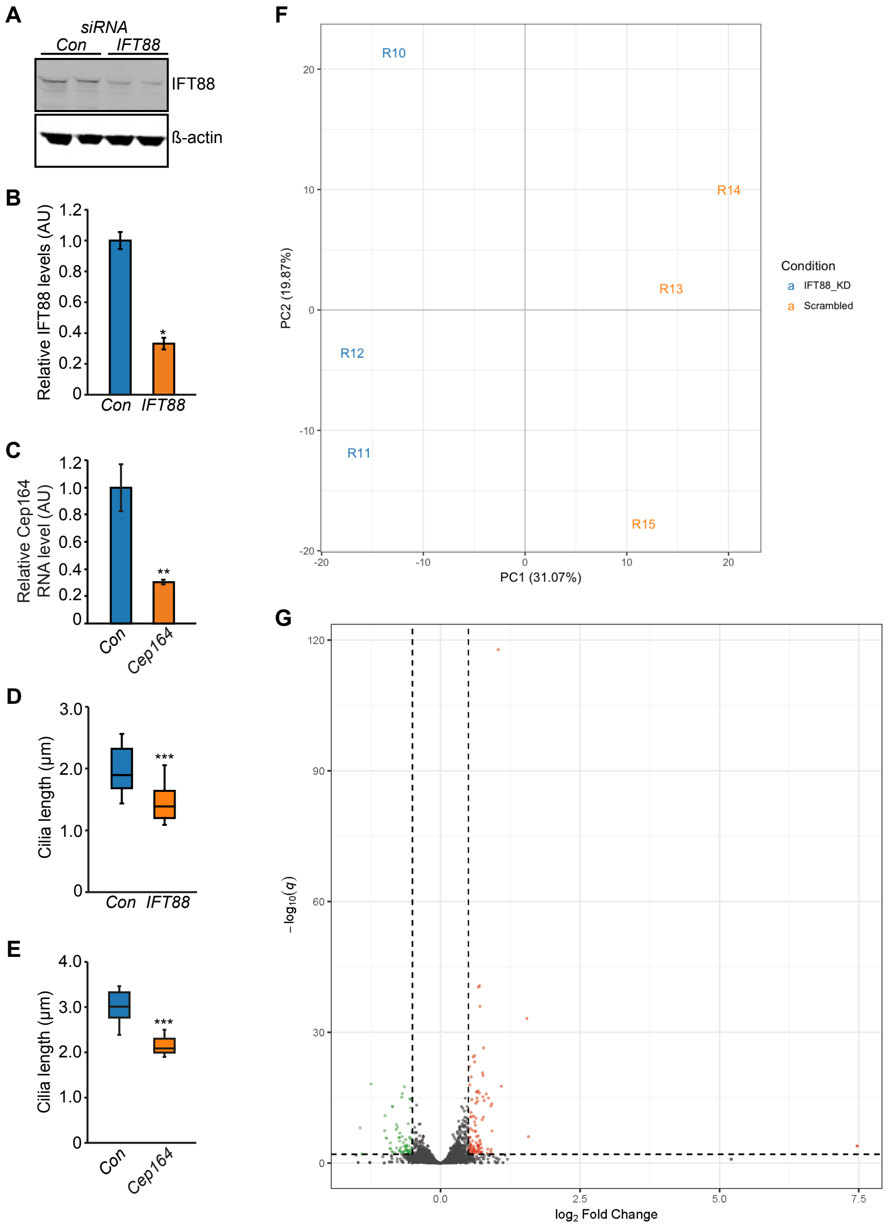


**Figure S3. Knockdown of cilia proteins and RNA-Seq comparison of the transcriptomes of PC12 cells transfected with siRNAs targeting IFT88 or non-targeting control siRNAs. (A)** Immunoblot of total cell lysates from PC12 cells transfected with siRNAs targeting IFT88 or non-targeting control siRNAs (Con). Cell lysates were generated 48 hours after transfection and immunoblots probed with an anti-IFT88 antibody and anti- β-actin as a loading control. **(B)** Densitometric analyses were performed and mean relative IFT88 protein levels calculated (n=6). Data were normalised to β-actin. **(C)** Relative RNA expression of Cep164 in PC12 cells 48 hours after they were transfected with siRNAs targeting this transcript. Cep164 levels are expressed relative to those in cells transfected with a non-targeting control siRNAs (arbitrary units). Data was normalized to levels of peptidylpropyl isomerase A. **(D, E)** Quantification of axoneme length from PC12 cells transfected with siRNAs targeting IFT88 **(D)** or Cep164 **(E)**, compared to cells transfected with non-targeting control siRNAs. 48 hours after transfection. Error bars indicate 2x SEM. Statistical tests: t-test. * P<0.05, **P<0.01, ***P<0.001. **(F)** Principal component analysis (PCA) of RNA-seq expression data from IFT88 knockdown (R10-R12) and control cells (R13-R15; n=3 per condition). **(G)** Volcano plot showing log10 FDR-adjusted q-values versus log2 fold change between IFT88 knockdown and control cells. The vertical and horizontal dotted lines indicate 2x or -2x fold change and P = 0.01, respectively.
