# Supplementary material for: Oncometabolite induced primary cilia loss in pheochromocytoma
Source: Endocr Relat Cancer. 2018 Sep 5;26(1):165–80. doi: 10.1530/ERC-18-0134 (PMC6215910; doi:10.1530/ERC-18-0134)
Supplement: Supporting Figure 4 [file erc-26-165-s004.pdf]

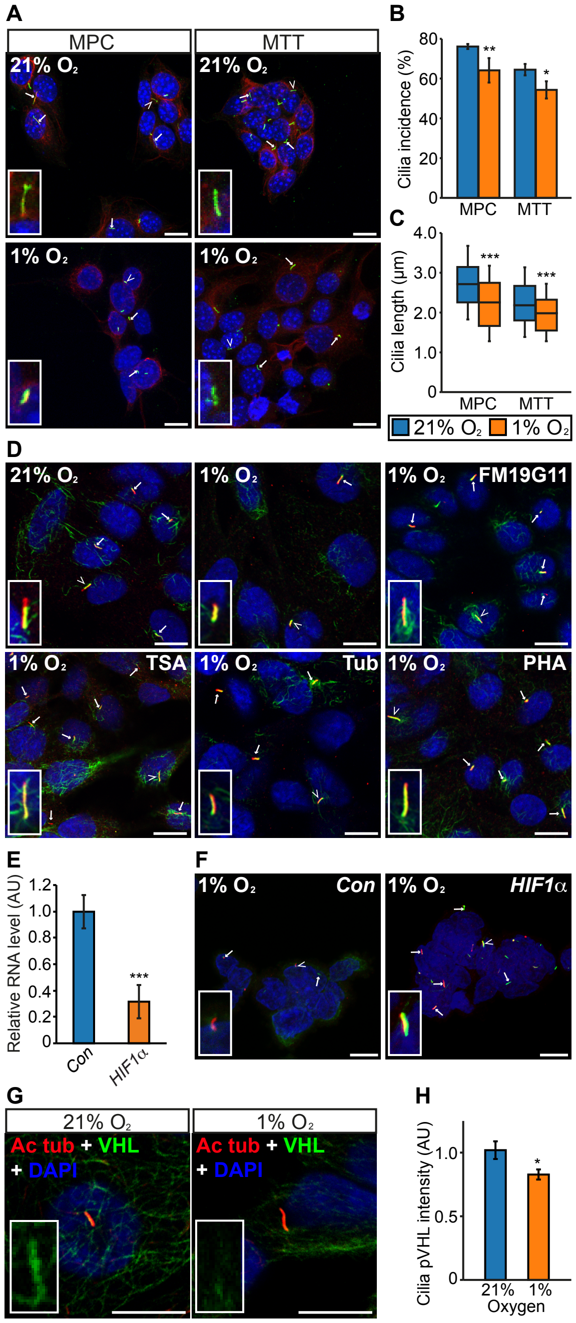

Figure S4. Primary cilia are lost from PCC derived cells when oxygen levels are reduced, while inhibition of both the Aurora-A/HDAC6 cilia resorption pathways and hypoxic signalling prevents cilia loss in cells cultured in 1% oxygen. (A) Confocal images of MPC and MTT cells cultured in 21% or 1% oxygen for 24 hours, prior to immunolabelling for detection of primary cilia. **(B, C)** Quantification of primary cilia incidence **(B)** and axonemal length **(C)** in MPC and MTT cells after 24 hours of culture in 21% and 1% oxygen. **(D)** Confocal images of PC12 cells immunolabelled for detection of primary cilia. Cells were cultured in 21% or 1% oxygen for 24 hours in the presence or absence of the inhibitors FM19G11, TSA, tubacin (Tub) and PHA-680632 (PHA), or vehicle only controls. **(E)** Relative RNA expression of HIF1α in PC12 cells 48 hours after they were transfected with siRNAs targeting this transcript. HIF1α levels are expressed relative to those in cells transfected with non-targeting control siRNAs (arbitrary units). Data was normalized to levels of peptidylpropyl isomerase A. **(F)** Confocal images of PC12 cells immunolabelled for detection of primary cilia. Cells were cultured in 1% oxygen after transfection with siRNAs targeting HIF1α or non-targeting control siRNAs, prior to immunolabelling for detection of primary cilia. **(G)** Confocal images of serum PC12 cells that were cultured in 21% or 1% oxygen for 24 hours prior to fixation and immunolabelling for detection of primary cilia (anti-acetylated α-tubulin, red) and pVHL (green). **(H)** Quantification of the intensity of pVHL staining in axonemes, relative to acetylated α-tubulin in ciliated cells that had been cultured in 21% or 1% oxygen. Scale bars = 10µm.
