# Supplementary material for: Oncometabolite induced primary cilia loss in pheochromocytoma
Source: Endocr Relat Cancer. 2018 Sep 5;26(1):165–80. doi: 10.1530/ERC-18-0134 (PMC6215910; doi:10.1530/ERC-18-0134)
Supplement: Supporting Figure 5 [file erc-26-165-s005.pdf]

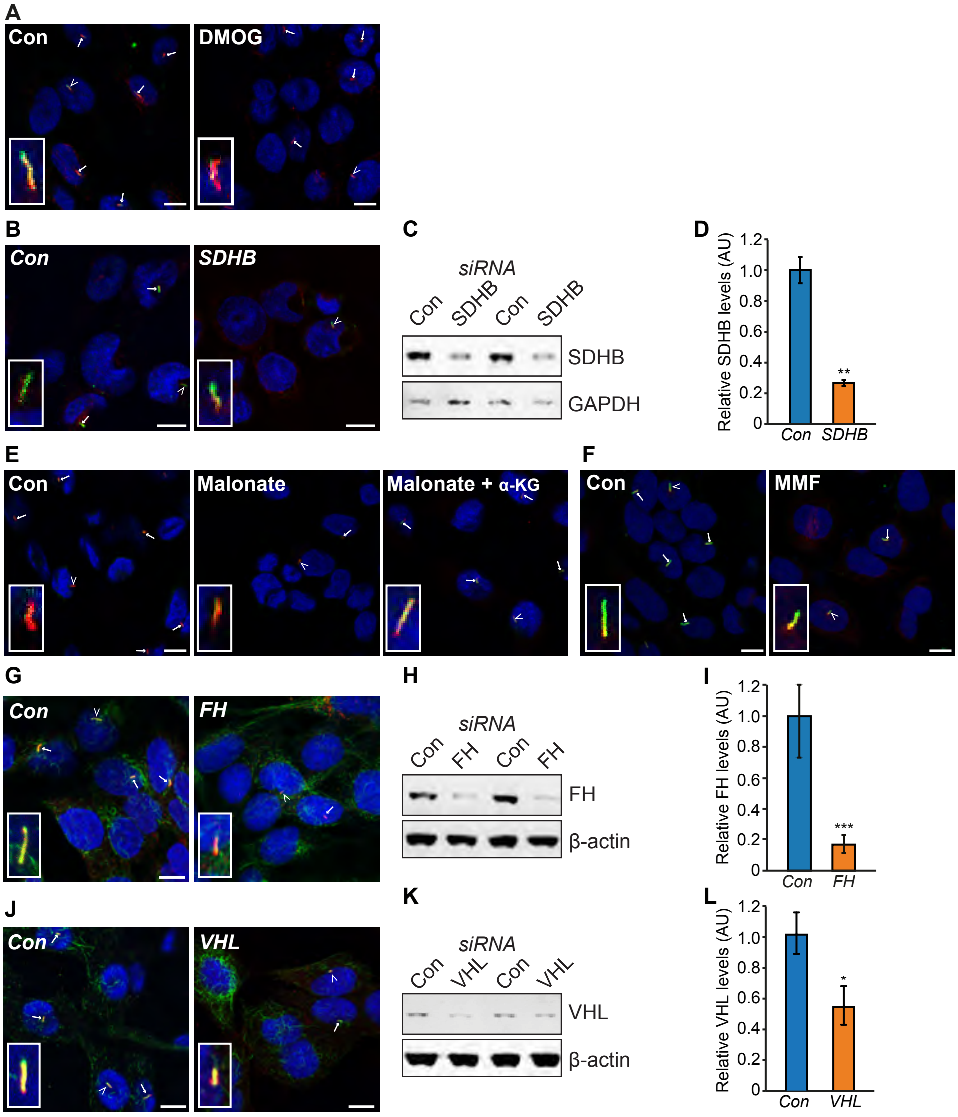

Figure S5. Primary cilia are lost from PC12 cells when the function of SDH, FH or VHL is targeted. (A) Confocal images of PC12 cells immunolabelled for detection of primary cilia after 24 hours treatment with DMOG. **(B)** Confocal images of PC12 cells immunolabelled for detection of primary cilia following siRNA mediated knockdown of SDHB. **(C, D)** Immunoblot **(C)** and densitometric analyses **(D)** of total cell lysates from PC12 cells transfected with siRNAs targeting SDHB or non-targeting control siRNAs (Con) and probed with antibodies against SDHB and GAPDH as a loading control. Data were normalised to GAPDH (n=6). **(E)** Confocal images of PC12 cells immunolabelled for detection of primary cilia after 24 hours treatment with malonate alone or with α-ketoglutarate (α-KG) **(F)** Confocal images of PC12 cells immunolabelled for detection of primary cilia after 24 hours treatment with monomethyl fumarate (MMF). **(G)** Confocal images of PC12 cells immunolabelled for detection of primary cilia following siRNA mediated knockdown of FH. **(H, I)** Immunoblot **(H)** and densitometric analyses **(I)** of total cell lysates from PC12 cells transfected with siRNAs targeting FH or non-targeting control siRNAs (Con) and probed with antibodies against FH and β-actin as a loading control. Data were normalised to β-actin (n=6). **(J)** Confocal images of PC12 cells immunolabelled for detection of primary cilia following siRNA mediated knockdown of VHL. **(K, L)** Immunoblot **(K)** and densitometric analyses **(L)** of total cell lysates from PC12 cells transfected with siRNAs targeting VHL or non-targeting control siRNAs (Con) and probed with antibodies against VHL and β-actin as a loading control. Data were normalised to β-actin (n=6). Scale bars = 10μm. Error bars indicate 2x SEM. Statistical tests used: t-test (C, H, K). * $P<0.05$, ** $P<0.01$, *** $P<0.001$.
