# Supplementary material for: Oncometabolite induced primary cilia loss in pheochromocytoma
Source: Endocr Relat Cancer. 2018 Sep 5;26(1):165–80. doi: 10.1530/ERC-18-0134 (PMC6215910; doi:10.1530/ERC-18-0134)
Supplement: Supporting Figure 6 [file erc-26-165-s006.pdf]

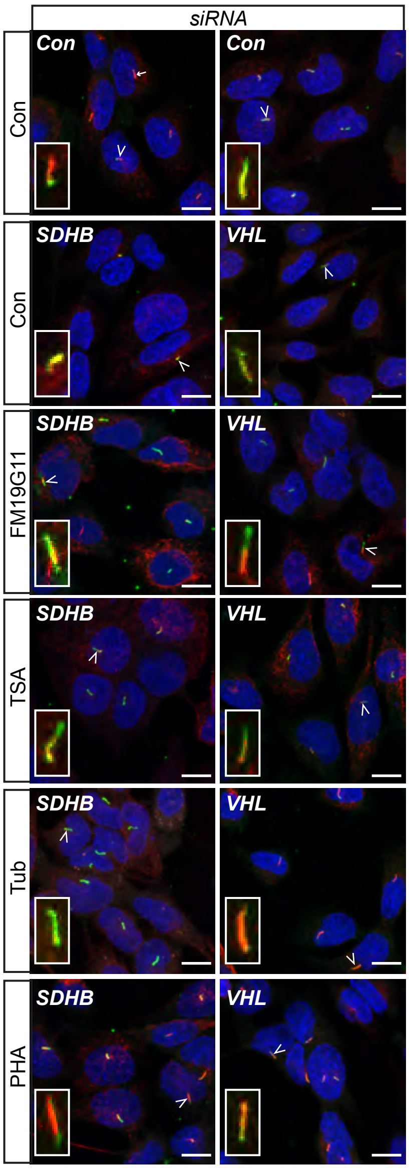

Figure S6. Confocal imaging shows inhibition of both the Aurora-A/HDAC6 cilia resorption pathways and of hypoxic signalling prevents cilia loss in SDHB and VHL knockdown cells. Confocal images of PC12 cells 48 hours after transfection with siRNAs targeting SDHB or VHL in the presence or absence of the inhibitors FM19G11, TSA, tubacin (Tub) and PHA-680632 (PHA), or vehicle only controls. Cells transfected with non-targeting control siRNAs (*Con*) were treated with the same inhibitors. Scale bar = 10μm.
